# Risk Factors for Grade 3 to Grade 4 Adverse Reactions to the ChAdOx1 nCoV-19 Vaccine (AZD1222) Against SARS-CoV-2

**DOI:** 10.3389/fmed.2021.738049

**Published:** 2021-09-30

**Authors:** Sang Won Lee, Hyun Lee, Sun-Kyung Lee, Ji-Yong Moon, SeolHwa Moon, Sung Jun Chung, Yoomi Yeo, Tai Sun Park, Dong Won Park, Tae-Hyung Kim, Jang Won Sohn, Ho Joo Yoon, Sang-Heon Kim

**Affiliations:** ^1^Department of Clinical Pharmacology and Therapeutics, Hanyang University Hospital, Seoul, South Korea; ^2^Division of Pulmonary Medicine and Allergy, Department of Internal Medicine, Hanyang University College of Medicine, Seoul, South Korea; ^3^Department of Mathematics, College of Natural Sciences, Hanyang University, Seoul, South Korea; ^4^College of Nursing, Hanyang University, Seoul, South Korea

**Keywords:** adverse reactions, ChAdOx1 nCoV-19 vaccine, coronavirus disease 2019, risk factors, healthcare workers

## Abstract

**Objective:** Limited data are available regarding the rates and risk factors of severe to serious adverse reactions (ARs) to the ChAdOx1 nCoV-19 vaccine.

**Methods:** Eligible participants were healthcare workers who received their first dose of the ChAdOx1 nCoV-19 vaccine in either of two university hospitals in Seoul, Korea. We evaluated the type and severity of ARs 7 days after the first dose of the ChAdOx1 nCoV-19 vaccine using a questionnaire survey delivered *via* a smartphone application link.

**Results:** Among the 1,603 participants who completed the survey, 684 (42.7%) participants experienced any kind of grade 3 to grade 4 AR. Being young (adjusted odds ratio [OR] for age 21–30 years = 2.49, 95% confidence interval [CI] = 1.75–3.56; adjusted OR for 31–40 years = 1.78, 95% CI = 1.22–2.62; adjusted OR for 41–50 years = 1.47, 95% CI = 1.03–2.11), being female (adjusted OR = 2.16. 95% CI = 1.62–2.89), and being underweight (adjusted OR = 1.61, 95% CI = 1.02–2.55) were identified as risk factors for grade 3 to grade 4 ARs. Among comorbidities, only diabetes mellitus (adjusted OR = 2.36, 95% CI = 1.03–5.53) was identified as a risk factor. When stratified by the type of AR, being young and being female were risk factors for both local and systemic grade 3 to grade 4 ARs.

**Conclusions:** Being young, female, or underweight and having diabetes mellitus were associated with an increased risk of developing grade 3 to grade 4 ARs after receiving the first dose of the ChAdOx1 nCoV-19 vaccine.

## Introduction

The SARS-CoV-2-induced coronavirus disease 2019 (COVID-19) pandemic remains a global health crisis. Vaccines against SARS-CoV-2 are regarded as the most powerful way to protect people from COVID-19. So far, various vaccines against SARS-CoV-2 have been developed using different technologies, and they have been approved by regulatory authorities based on efficacy and safety data from clinical trials (1). However, since the initiation of vaccination programs in many countries, concerns have grown about adverse reactions (ARs) to COVID-19 vaccines, particularly the ChAdOx1 nCoV-19 vaccine (AZD1222) (2–4).

The ChAdOx1 nCoV-19 vaccine is a chimpanzee adenovirus-vectored vaccine that encodes the full-length SARS-CoV-2 spike protein. Safety data about the ChAdOx1 nCoV-19 vaccine were based on a pooled analysis of data from clinical trials (5–8) that enrolled a mostly white population, with only a small representation from a few minority ethnic groups. It was reported that most local or systemic ARs were mild to moderate in severity, and the overall rate of ARs was lower in older adults than in younger ones (6). Recent reports from the real world, on the other hand, suggest that the rate of ARs to the ChAdOx1 nCoV-19 vaccine is higher than that to the BNT162b2 mRNA vaccine (9–11), though the ARs to the ChAdOx1 nCoV-19 are less frequent in the older age group (12). However, the rates and risk factors of severe to serious ARs to the ChAdOx1 nCoV-19 vaccine have not yet been reported.

We performed a prospective observational study to assess the safety of the ChAdOx1 nCoV-19 vaccine among healthcare workers (HCWs) at two university hospitals in Korea. Here, we report the risk factors associated with the development of grade 3 to grade 4 ARs.

## Methods

### Study Population and Study Design

Eligible participants were HCWs (physicians, nurses, laboratory technicians, and administrative workers) who received their first dose of ChAdOx1 nCoV-19 vaccine between March 8, 2021, and March 11, 2021, at Hanyang University Hospital or Hanyang University Guri Hospital, Seoul, Korea. Participants were included only after they signed the voluntary informed consent form. This study was approved by the Institutional Review Board of Hanyang University Hospital (IRB number: HYUH 2021-02-029-002).

Seven days after their first dose of ChAdOx1 nCoV-19, participants were asked to complete an AR online survey, prompted by a text message that contained a smartphone application link. To increase the response rate among the participants, additional notification messages were sent to participants who did not respond to the initial request.

### Adverse Reactions Survey

All participants received the same set of questions. The questions consisted of two major categories: baseline characteristics and ARs. The baseline characteristics were age, sex, height, weight, occupation (physician, nurse, laboratory technician, or administrative worker), history of allergies, history of COVID-19 infection, comorbidities, and medication history. Body mass index was calculated by dividing participants' weight by the square of their height and was categorized as underweight (<18.5 kg/m^2^), normal (18.5–22.9 kg/m^2^), overweight (23.0–24.9 kg/m^2^), and obese (≥25.0 kg/m^2^), according to the Asian-Pacific definitions of obesity (13).

The solicited ARs included systemic (i.e., fever, fatigue, chills, headache, muscle aches, joint pain, loss of appetite, diarrhea, vomiting, constipation, stomachache, rash, dizziness, cough, dyspnea, and hypersensitivity) and local reactions (i.e., pain, tenderness, redness, induration, urticaria, and itching). The severity of the ARs was grouped into four categories (grade 1, mild; grade 2, moderate; grade 3, severe; grade 4, potentially life threatening) which were based on the Food and Drug Administration guidelines (See Supplementary Tables 2, 3 for detailed classification of grade 1 to 4 for each AR) (14). Data regarding time to onset (within 30 min, 24 h, 3 or 7 days), treatments and use of healthcare to manage adverse reactions (e.g., medications, emergency department visit, or hospitalization), and resolution of the ARs were also collected. To cover more topics without increasing participants' burden, most questions and answers were constructed using a pre-defined choice format.

### Aim of the Study

This was a prospective observational study among HCWs to evaluate the risk factors for grade 3 to grade 4 ARs to ChAdOx1 nCoV-19 vaccination.

### Statistical Analyses

Continuous variables are presented as means ± SD and were compared using Student's *t*-testing or Mann–Whitney *U*-testing, as appropriate. Categorical variables are presented as numbers (%) and were compared using the chi-squared test or Fisher's exact test, as appropriate. A *p* < 0.05 was considered statistically significant. To find the risk factors for grade 3 to grade 4 adverse reactions, odds ratios (ORs) were evaluated using univariable and multivariable logistic regression analyses. Results are summarized using ORs and 95% confidence intervals (CIs). All statistical analyses were performed in SAS® 9.4 (SAS Institute, Cary, NC, USA).

## Results

### Study Population

A total of 2,235 participants received the ChAdOx1 nCoV-19 vaccine and gave their informed consent. Among them, 1,603 participants completed the survey, yielding a response rate of 71.7%. The mean age was 37.7 ± 10.8 years, and 1,261 (78.7%) participants were female. The mean body mass index (BMI) was 22.5 ± 3.9 kg/m^2^. Regarding occupation, 12.1% were physicians, 60.8% were nurses, 11.4% were laboratory technicians, and 15.7% were administrative workers. Only two participants reported having a history of COVID-19 infection. The most common comorbidities reported were allergies other than asthma (4.6%), hypertension (3.1%), and diabetes mellitus (1.7%) (Table 1).

**Table 1:** Baseline characteristics of the study population.

	**Total**	**Without, grade 1, or grade 2 AR**	**Grade 3 to grade 4 AR**	***P*-value**
	**(*n* = 1,603)**	**(*n* = 919)**	**(*n* = 684)**	
**Age, years**	37.7 ± 10.8	39.5 ± 11.0	35.4 ± 10.1	<0.001
21–30	588 (36.7)	275 (29.9)	313 (45.8)	<0.001
31–40	319 (19.9)	189 (20.6)	130 (19.0)	
41–50	468 (29.2)	290 (31.6)	178 (26.0)	
51–60	214 (13.4)	152 (16.5)	62 (9.1)	
>60	14 (0.9)	13 (1.4)	1 (0.2)	
**Female**	1,261 (78.7)	664 (72.3)	597 (87.3)	<0.001
**Body mass index, kg/m** ^**2**^	22.5 ± 3.3	22.9 ± 3.3	22.0 ± 3.2	<0.001
<18.5	121 (7.6)	51 (5.6)	70 (10.2)	
18.5–22.9	867 (54.1)	465 (50.6)	402 (58.8)	
23.0–24.9	292 (18.2)	187 (20.4)	105 (15.4)	
≥25	323 (20.2)	216 (23.5)	107 (15.6)	
**Occupation**				<0.001
Physician	194 (12.1)	123 (13.4)	71 (10.4)	
Nurse	975 (60.8)	500 (54.4)	475 (69.4)	
Laboratory technician	183 (11.4)	120 (13.1)	63 (9.2)	
Administrative worker	251 (15.7)	176 (19.2)	75 (11.0)	
**Comorbidities**				
Any allergy other than asthma	74 (4.6)	34 (3.7)	40 (5.9)	0.057
Hypertension	50 (3.1)	29 (3.2)	21 (3.1)	0.923
Diabetes mellitus	27 (1.7)	12 (1.3)	15 (2.2)	0.172
Asthma	10 (0.6)	5 (0.5)	5 (0.7)	0.752
Chronic liver disease	9 (0.6)	8 (0.9)	1 (0.2)	0.087
Chronic heart disease	6 (0.4)	5 (0.5)	1 (0.2)	0.248
Chronic pulmonary disease	3 (0.19)	2 (0.2)	1 (0.2)	>0.999
Other comorbidities	92 (5.7)	56 (6.1)	36 (5.3)	0.480

*Data are presented as frequency (%) and mean with standard deviation*.

### Rate of Adverse Reactions

**Figure 1:**
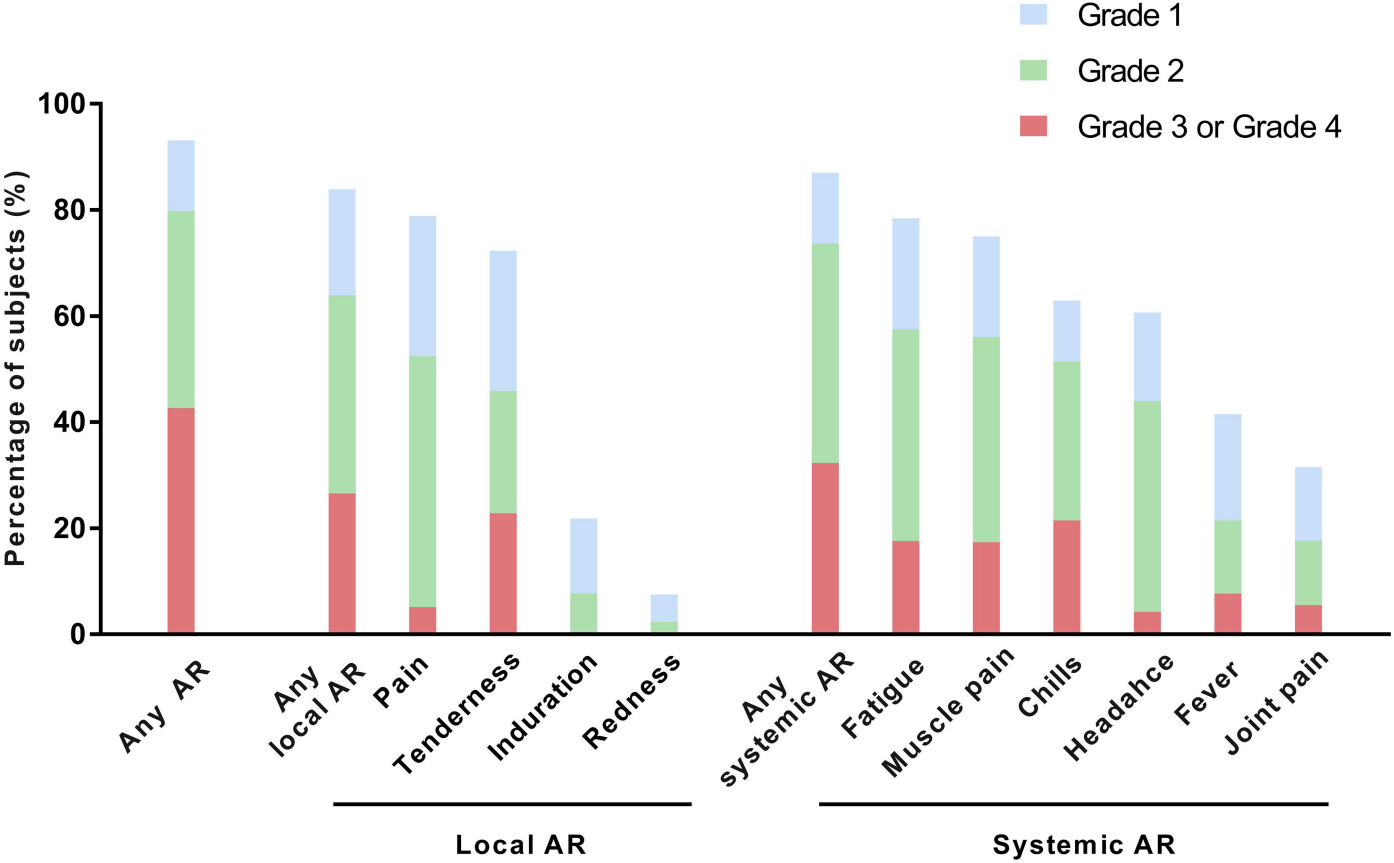
Solicited adverse reactions after the first dose of ChAdOx1 nCoV-19. AR, adverse reaction.

Among the 1,603 participants who responded to the survey, 1,493 (93.1%) participants reported experiencing an AR, and 684 (42.7%) participants reported experiencing a grade 3 to grade 4 AR. The rates of HCWs who reported systemic ARs and local ARs were similar (87.0 vs. 84.0%); however, grade 3 to grade 4 ARs were more frequently reported for systemic ARs than for local ARs (32.3 vs. 26.6%). The most frequently reported AR was pain (78.9%), followed by fatigue (78.4%), muscle pain (75.1%), and chills (62.9%). However, the most frequently reported grade 3 to grade 4 AR was tenderness (22.8%; grade 3, 22.4%; grade 4, 0.4%), followed by chills (21.5%; grade 3, 20%; grade 4, 1.5%), fatigue (17.7%; grade 3, 16.6%, grade 4, 1.1%), and muscle pain (17.4%; grade 3, 16.3%; grade 4, 1.1%; Figure 1).

### Factors Influencing the Development of Grade 3 to Grade 4 Adverse Reactions

In the overall study population, age, sex, and BMI were identified as risk factors for grade 3 to grade 4 ARs in the multivariate logistic regression model. The younger age group (21–50 years) (adjusted OR for aged 21–30 years = 2.49, 95% CI = 1.75–3.56; adjusted OR for aged 31–40 years = 1.78, 95% CI = 1.22–2.62; adjusted OR = aged 41–50 years = 1.47), females (adjusted OR for female = 2.16, 95% CI = 1.62–2.89), and the underweight group (< 18.5 kg/m^2^) (adjusted OR = 1.61 95% CI 1.02–2.55) had a higher risk of developing grade 3 to grade 4 ARs than the older age group (>50 years), males, and the normal or higher BMI groups (≥18.5 kg/m^2^), respectively. Comorbidities other than diabetes mellitus (adjusted OR = 2.36, 95% CI = 1.03–5.53), did not play a critical role in developing grade 3 to grade 4 ARs (Table 2).

**Table 2:** Factors associated with overall grade 3 to grade 4 adverse reactions^*^.

	**Crude model**	**Adjusted model**
**Age group, years**		
21–30	2.98 (2.15–4.18)	2.49 (1.75–3.56)
31–40	1.80 (1.25–2.61)	1.78 (1.22–2.62)
41–50	1.61 (1.14–2.28)	1.47 (1.03–2.11)
>50	Reference	Reference
**Female**	2.64 (2.03–3.46)	2.16 (1.62–2.89)
**Body mass index, kg/m** ^**2**^		
<18.5	2.77 (1.81–4.27)	1.61 (1.02–2.55)
18.5–22.9	1.75 (1.34–2.29)	1.24 (0.93–1.66)
23.0–24.9	1.13 (0.81–1.58)	1.09 (0.77–1.53)
≥25	Reference	Reference
**Comorbidities**		
Any allergy other than asthma	1.72 (0.94–3.21)	1.45 (0.77–2.79)
Hypertension	0.97 (0.54–1.71)	1.16 (0.61–2.16)
Diabetes mellitus	1.70 (0.79–3.72)	2.36 (1.03–5.53)
Asthma	1.35 (0.37–4.86)	1.27 (0.33–4.94)
Chronic liver disease	0.17 (0.01–0.91)	0.19 (0.01–1.11)
Chronic heart disease	0.27 (0.01–1.67)	0.26 (0.01–1.70)
Chronic pulmonary disease	0.67 (0.03–7.02)	0.57 (0.03–6.48)
Other comorbidities	0.86 (0.55–1.31)	1.08 (0.69–1.69)

*Data are presented as odds ratio (95% confidence interval)*.

* *These reactions include any grade 3 to grade 4 systemic or local reactions*.

Next, we performed subgroup analyses according to the type of AR and sex. For both local and systemic ARs, being female was identified as a risk factor for developing grade 3 to grade 4 ARs (adjusted OR for local ARs = 2.71, 95% CI = 1.91–3.93; adjusted OR for systemic ARs = 1.94, 95% CI = 1.42–2.97). Age was identified as a risk factor for developing grade 3 to grade 4 systemic ARs (adjusted OR for aged 21–30 years = 3.70, 95% CI = 2.49–5.63; adjusted OR for aged 31–40 years = 2.26, 95% CI = 1.47–3.54; adjusted OR for aged 41–50 years = 1.61, 95% CI = 1.06–2.47), but not for developing local ARs (Table 3).

**Table 3:** Factors associated with grade 3 to grade 4 local and systemic adverse reactions.

	**Grade 3 to grade 4 local reactions**		**Grade 3 to grade 4 systemic reactions**	
	**Crude model**	**Adjusted model**	**Crude model**	**Adjusted model**
**Age group, years**				
21–30	1.62 (1.13–2.35)	1.40 (0.95–2.08)	4.29 (2.95–6.41)	3.70 (2.49–5.63)
31–40	1.25 (0.83–1.89)	1.23 (0.81–1.90)	2.26 (1.49–3.48)	2.26 (1.47–3.54)
41–50	1.43 (0.99–2.11)	1.31 (0.89–1.95)	1.72 (1.15–2.62)	1.61 (1.06–2.47)
>50	Reference	Reference	Reference	Reference
**Female**	2.94 (2.12–4.18)	2.71 (1.91–3.93)	2.44 (1.83–3.31)	1.94 (1.42–2.70)
**Body mass index, kg/m** ^**2**^				
<18.5	1.35 (0.84–2.14)	0.93 (0.56–1.54)	2.99 (1.93–4.64)	1.56 (0.97–2.51)
18.5–22.9	1.39 (1.03–1.87)	1.05 (0.77–1.45)	1.69 (1.27–2.27)	1.16 (0.85–1.60)
23.0–24.9	0.86 (0.58–1.25)	0.81 (0.55–1.20)	1.15 (0.80–1.65)	1.10 (0.75–1.60)
≥25.0	Reference	Reference	Reference	Reference
**Comorbidities**				
Any allergy other than asthma	1.50 (0.77–2.79)	1.45 (0.73–2.76)	1.85 (1.00–3.41)	1.52 (0.79–2.90)
Hypertension	1.44 (0.78–2.58)	1.62 (0.83–3.05)	1.08 (0.58–1.93)	1.56 (0.80–2.98)
Diabetes mellitus	1.39 (0.59–3.04)	1.45 (0.58–3.40)	1.05 (0.45–2.29)	1.34 (0.54–3.14)
Asthma	0.69 (0.10–2.76)	0.56 (0.08–2.39)	1.40 (0.36–4.92)	1.39 (0.32–5.44)
Chronic liver disease	–	–	0.26 (0.01–1.43)	0.29 (0.02–1.72)
Chronic heart disease	–	–	0.42 (0.02–2.60)	0.39 (0.02–2.51)
Chronic pulmonary disease	1.38 (0.06–14.46)	1.30 (0.06–13.69)	–	–
Other comorbidities	1.16 (0.72–1.82)	1.31 (0.80–2.10)	0.73 (0.44–1.16)	0.99 (0.59–1.61)

*Data are presented as odds ratio (95% confidence interval)*.

When we stratified the population by sex, age was a risk factor of developing grade 3 to grade 4 ARs for both females (adjusted OR for aged 21–30 years = 2.37, 95% CI = 1.59–3.57; adjusted OR for aged 31–40 years = 1.56, 95% CI = 1.00–2.44; adjusted OR for aged 41–50 years = 1.44, 95% CI = 0.96–2.17) and males (adjusted OR for aged 21–30 years = 2.92, 95% CI = 1.32–6.82; adjusted OR for aged 31–40 years = 2.95, 95% CI = 1.37–6.74; adjusted OR for aged 41–50 years = 1.41, 95% CI = 0.61–3.34). However, BMI <25.0 kg/m^2^ was significantly associated with an increased risk of grade 3 to grade 4 ARs only in females (adjusted OR for underweight = 2.09, 95% CI = 1.27–3.46; adjusted OR for normal = 1.58, 95% CI = 1.13–2.25; adjusted OR for overweight = 1.54, 95% CI = 1.01–2.37). Regarding comorbidities, diabetes mellitus was associated with an increased risk of grade 3 to grade 4 ARs in females (adjusted OR = 5.50, 95% CI = 1.81–20.73), and any allergy other than asthma (adjusted OR = 9.73, 95% CI = 1.07–210.61) and hypertension (adjusted OR = 4.02, 95% CI = 1.15–14.50) were associated with an increased risk of grade 3 to grade 4 ARs in males (Supplementary Table 1 and Supplementary Figures 1–3).

## Discussion

This study evaluated the rate of and risk factors associated with grade 3 to grade 4 ARs among HCWs who received their first dose of the ChAdOx1 nCoV-19 vaccine. We found that about 40% of HCWs had grade 3 to grade 4 ARs. Being young, female, or underweight and having diabetes mellitus were all significantly associated with an increased risk of developing grade 3 to grade 4 ARs. For young (aged 21–50) females with BMI < 18.5 kg/m^2^ (*n* = 115), the most frequent grade 3 to grade 4 adverse reaction was chills (systemic AR; 32.2%; grade 3, 28.7; grade 4, 3.5%) followed by fatigue (systemic AR; 26.9%; grade 3, 22.6%; grade 4, 4.3%), tenderness (local AR; 23.5%; grade 3, 23.5%; grade 4, 0%), muscle pain (systemic AR; 22.6%; grade 3, 20.9%; grade 4, 1.7%), and fever (systemic AR; 18.2%; grade 3, 16.5%; grade 4, 1.7%). In the stratified analyses by the type of AR, being female was solely associated with grade 3 to grade 4 local ARs, whereas younger age and female sex were associated with grade 3 to grade 4 systemic ARs. When we stratified the population by sex, young age, low BMI, and diabetes mellitus were risk factors for grade 3 to grade 4 ARs in females, and young age, allergic disease, and hypertension were risk factors for grade 3 to grade 4 ARs in males.

As reported in previous studies evaluating ARs to the ChAdOx1 nCoV-19 vaccine in Korean HCWs (9–12), more than 90% of HCWs in our study reported experiencing any AR. However, because previous studies focused on comparing ARs between HCWs who received the ChAdOx1 nCoV-19 vaccine and those who received the BNT162b2 mRNA COVID-19 vaccine (9–11), the rate of grade 3 to grade 4 ARs after the ChAdOx1 nCoV-19 vaccine has not been well-elucidated. Because we prospectively collected data about AR severity, our study provides valuable information about this research question: about 40% of the HCWs in our study population developed grade 3 to grade 4 ARs. The most common grade 3 to grade 4 local ARs were tenderness and chills, followed by fatigue and muscle pain. These results are interesting because previous studies emphasized fever by specifically describing the rate of fever. This disparity is thought to be caused by the difference between side effects that doctors consider important (e.g., fever, anaphylaxis) and the symptoms most seriously felt by those who receive the vaccine. Our study results show that more attention needs to be paid to controlling constitutional symptoms after the ChAdOx1 nCoV-19 vaccine.

The randomized controlled trial that evaluated the safety of the ChAdOx1 nCoV-19 vaccine provided information about ARs by age group. Ramasamy et al. (6) showed that severe ARs to the ChAdOx1 nCoV-19 vaccine occurred more commonly in people aged 18–55 years than in people older than that. A study evaluating the rate of ARs to the SARS-CoV-2 mRNA-1273 vaccine in the elderly population showed that people aged 56–70 years and those >70 years had similar AR rates, and no severe ARs occurred in this relatively old population (15). However, given that previous studies used 55 years as the cutoff to differentiate the young population from the elderly population (6, 15), it is unclear whether the rate of severe to serious ARs is affected by age among subjects younger than 55 years. Our study population was relatively young (about 85% was <50 years), allowing us to provide detailed information about that issue. We found a dose-dependent inverse relationship between age and grade 3 to grade 4 ARs, suggesting that age-related immune reactions to the ChAdOx1 nCoV-19 vaccine play a crucial role in the occurrence of grade 3 to grade 4 systemic ARs.

In recent observational studies (9, 11, 12), ARs to the ChAdOx1 nCoV-19 vaccine were more common among young people and females than among old people and males. However, those previous studies did not control for potential bias. We controlled for potential confounders, such as age, sex, BMI, and comorbidities, by adjustment or stratification. After controlling for those factors, we found that young age and being female are independent risk factors for developing grade 3 to grade 4 ARs to the ChAdOx1 nCoV-19 vaccine. When we stratified the data by the type of AR, being female was the only significant risk factor for the occurrence of grade 3 to grade 4 local ARs. In comparison, young age and being female were significantly associated with grade 3 to grade 4 systemic ARs. Furthermore, being underweight was significantly associated with an increased risk of grade 3 to grade 4 ARs to the ChAdOx1 nCoV-19 vaccine. The reasons that grade 3 to grade 4 ARs are more common among young people, females, and those with low BMI are not known. A recent study evaluating the antibody response to the BTN162b2 vaccine showed that titers of COVID-19-binding antibodies were higher in the population with those characteristics (16). Although no studies have evaluated the antibody response to the ChAdOx1 nCoV-19 vaccine, the enhanced immunogenicity in those populations could partly explain the increased ARs. It is also well-known that females are prone to allergic reactions, anaphylaxis, and cutaneous reactions after COVID-19 vaccination (17–19).

Interestingly, we found that diabetes mellitus increases the risk of grade 3 to grade 4 ARs, especially in females. It has been suggested that the immune response and antibody response to the influenza vaccine is similar between subjects with and without diabetes mellitus (20). However, as no studies are available regarding the association between the immunogenicity of the ChAdOx1 nCoV-19 vaccine and diabetes mellitus (21), further studies are needed. Allergic diseases and hypertension were related to grade 3 to grade 4 ARs in males but not in females. Nonetheless, as comorbidities are usually linked to demographics (e.g., age, sex, or BMI) in a complicated manner, it is difficult to provide plausible reasons for the phenomenon. Despite this limitation, our study is the first (to the best of our knowledge) to suggest that comorbid profiles could play a role in the occurrence of grade 3 to grade 4 ARs. Particular attention should be paid to subjects with these comorbid profiles after they receive the ChAdOx1 nCoV-19 vaccine.

Our study has several limitations. First, it was performed in a single center in Korea. The risk factors for grade 3 to grade 4 ARs could be different in populations in other countries. Second, we enrolled relatively healthy subjects working at hospitals, so our study population had a relatively low rate of comorbidities and might not represent the general population. The prevalence of comorbidities was also likely underestimated because the presence of comorbidities was based only on participant reports. Accordingly, careful interpretation is needed for the association between comorbidities and grade 3 to grade 4 ARs. Third, because we evaluated HCWs, the number of elderly subjects was relatively small. Thus, the safety of the vaccine among people older than 65 years could not be determined. Last, we did not analyze the association between a previous COVID-19 infection and the rate of ARs due to the small number of previous infection cases. Intriguingly, a recent study reported that a prior COVID-19 illness was associated with an increased risk of ARs to COVID-19 vaccines (22).

In conclusion, grade 3 to grade 4 ARs occurred in about 40% of HCWs. Young age, female sex, underweight status, and diabetes mellitus were significantly associated with an increased risk of developing grade 3 to grade 4 ARs.

## Data Availability Statement

The raw data supporting the conclusions of this article will be made available by the authors, without undue reservation.

## Ethics Statement

The studies involving human participants were reviewed and approved by Institutional Review Board of Hanyang University Hospital. The patients/participants provided their written informed consent to participate in this study.

## Author Contributions

SL, HL, J-YM, YY, TP, DW, T-HK, JS, HY, and S-HK contributed to the conception and design of the study. SM and SC organized the database. S-KL performed the statistical analysis. SL, HL, and S-HK wrote the first draft of the manuscript. SL, HL, S-KL, and S-HK wrote sections of the manuscript. All authors contributed to manuscript revision, read, and approved the submitted version.

## Funding

This research was supported by a grant from the Korean Academy of Asthma, Allergy and Clinical Immunology and the Korean Health Technology R&D Project through the Korea Health Industry Development Institute (KHIDI), funded by the Ministry of Health and Welfare, Republic of Korea (grant number: HI19C0218).

## Conflict of Interest

The authors declare that the research was conducted in the absence of any commercial or financial relationships that could be construed as a potential conflict of interest.

## Publisher's Note

All claims expressed in this article are solely those of the authors and do not necessarily represent those of their affiliated organizations, or those of the publisher, the editors and the reviewers. Any product that may be evaluated in this article, or claim that may be made by its manufacturer, is not guaranteed or endorsed by the publisher.
