# Supplementary material for: Risk Factors for Grade 3 to Grade 4 Adverse Reactions to the ChAdOx1 nCoV-19 Vaccine (AZD1222) Against SARS-CoV-2
Source: Front Med (Lausanne). 2021 Sep 30;8:738049. doi: 10.3389/fmed.2021.738049 (PMC8514770; doi:10.3389/fmed.2021.738049)
Supplement: Supplementary file 1 [file Table_1.DOCX]

**Supplementary Table 1.** **Factors associated with grade 3 to grade 4 adverse reactions by sex**

|  | **Females** | | **Males** | |
| --- | --- | --- | --- | --- |
|  | **Crude model** | **Adjusted model** | **Crude model** | **Adjusted model** |
| **Age group, years** |  |  |  |  |
| 21-30 | 2.49 (1.70–3.69) | 2.37 (1.59–3.57) | 2.45 (1.18–5.28) | 2.92 (1.32–6.82) |
| 31-40 | 1.54 (1.00–2.38) | 1.56 (1.00–2.44) | 2.38 (1.18–5.01) | 2.95 (1.37–6.74) |
| 41-50 | 1.42 (0.95–2.13) | 1.44 (0.96–2.17) | 1.27 (0.59–2.78) | 1.41 (0.61–3.34) |
| >50 | Reference | Reference | Reference | Reference |
| **Body mass index, kg/m^2^** |  |  |  |  |
| <18.5 | 2.59 (1.62–4.19) | 2.09 (1.27–3.46) | 0.79 (0.04–6.36) | 0.66 (0.03–5.48) |
| 18.5-22.9 | 1.72 (1.24–2.41) | 1.58 (1.13–2.25) | 0.82 (0.45–1.48) | 0.81 (0.43–1.50) |
| 23.0-24.9 | 1.50 (0.99–2.28) | 1.54 (1.01–2.37) | 0.58 (0.31–1.04) | 0.65 (0.34–1.21) |
| ≥25.0 | Reference | Reference | Reference | Reference |
| **Comorbidities** |  |  |  |  |
| Any allergy other than asthma | 1.31 (0.69–2.51) | 1.18 (0.60–2.31) | 9.06 (1.15–184.67) | 9.73 (1.07–210.61) |
| Hypertension | 0.74 (0.36–1.45) | 0.73 (0.33–1.53) | 2.70 (0.92–7.76) | 4.02 (1.15–14.50) |
| Diabetes mellitus | 3.67 (1.29–13.10) | 5.50 (1.81–20.73) | 0.73 (0.11–2.97) | 0.49 (0.06–2.42) |
| Asthma | 1.39 (0.37–5.65) | 1.61 (0.40–6.88) | - | - |
| Chronic liver disease | 0.28 (0.01–1.88) | 0.25 (0.01–1.74) | - | - |
| Chronic heart disease | - | - | 2.95 (0.12–75.16) | 1.87 (0.07–48.09) |
| Chronic pulmonary disease | 0.56 (0.03–5.81) | 0.60 (0.03–7.06) | - | - |
| Other comorbidities | 1.03 (0.64–1.65) | 1.33 (0.81–2.18) | 0.15 (0.01–0.76) | 0.19 (0.01–1.00) |

Data are presented as odds ratio (95% confidence interval).
