# Supplementary material for: Risk Factors for Grade 3 to Grade 4 Adverse Reactions to the ChAdOx1 nCoV-19 Vaccine (AZD1222) Against SARS-CoV-2
Source: Front Med (Lausanne). 2021 Sep 30;8:738049. doi: 10.3389/fmed.2021.738049 (PMC8514770; doi:10.3389/fmed.2021.738049)
Supplement: Supplementary file 2 [file Table_2.DOCX]

**Supplementary Table 2.** **Grading criteria for local adverse reactions**

| **Local adverse reactions** | **Mild  (Grade 1)** | **Moderate  (Grade 2)** | **Severe  (Grade 3)** | **Potentially Life Threatening  (Grade 4)** |
| --- | --- | --- | --- | --- |
| Pain | Dose not interfere with activity | Repeated use of non-narcotic pain reliever > 24 hours or interferes with activity | Any use of narcotic pain reliever or prevents daily activity | Emergency room visit or hospitalization |
| Tenderness | Mild discomfort to touch | Discomfort with movement | Significant discomfort at rest | Emergency room visit or hospitalization |
| Erythema/Redness | 2.5-5 cm | 5.1-10 cm | >10 cm | Necrosis or exfoliative dermatitis |
| Induration/Swelling | 2.5-5 cm and does not interfere with activity | 5.1-10 cm or interferes with activity | >10 cm or prevents daily activity | Necrosis |
| Urticaria | No need for medication | Use of oral or local or intravenous medication or steroid treatment < 24 hours | Use of intravenous medication or steroid treatment > 24 hours | - |
| Itch | Mild itch at site of administration | Significant itch at limbs that were not administered | Itch at administered limb and other parts of body | Itch at entire body |
| Other | No interference with activity | Some interference with activity not requiring medical intervention | Prevents daily activity and requires medical intervention | Emergency room visit or hospitalization |

Grading criteria defined based on the Food and Drug Administration guideline.^14^
