# Supplementary material for: Risk Factors for Grade 3 to Grade 4 Adverse Reactions to the ChAdOx1 nCoV-19 Vaccine (AZD1222) Against SARS-CoV-2
Source: Front Med (Lausanne). 2021 Sep 30;8:738049. doi: 10.3389/fmed.2021.738049 (PMC8514770; doi:10.3389/fmed.2021.738049)
Supplement: Supplementary file 3 [file Table_3.DOCX]

**Supplementary Table 3.** **Grading criteria for systemic adverse reactions**

| **Systemic adverse reactions** | **Mild  (Grade 1)** | **Moderate  (Grade 2)** | **Severe  (Grade 3)** | **Potentially Life Threatening  (Grade 4)** |
| --- | --- | --- | --- | --- |
| Fever | 38.0-38.4°C | 38.5-38.9°C | 39.0-40°C | > 40°C |
| Fatigue | No interference with activity | Some interference with activity | Significant; prevents daily activity | Emergency room visit or hospitalization |
| Chills | No interference with activity | Some interference with activity | Significant; prevents daily activity | Emergency room visit or hospitalization |
| Headache | No interference with activity | Repeated use of non-narcotic pain reliever > 24 hours or some interference with activity | Significant; any use of narcotic pain reliever or prevents daily activity | Emergency room visit or hospitalization |
| Muscle pain | No interference with activity | Some interference with activity | Significant; prevents daily activity | Emergency room visit or hospitalization |
| Joint pain | Mild pain with no interference with activity | Moderate pain that has some interference with activity but does not prevent daily activity (inflammation, redness or joint swelling accompanied) | Significant pain; prevents daily activity (inflammation, redness or joint swelling accompanied) | Emergency room visit or hospitalization |
| Loss of appetite | Decreased food intake, decreased oral intake < 48 hours | Decreased appetite, interference with activity, decreased oral intake > 48 hours with no significant weight loss | Significantly decreased appetite, significant weight loss | - |
| Diarrhea | 2-3 loose stools or < 400 g/24 hours | 4-5 stools or 400-500 g/24 hours | 6 or more watery stools or > 800 g/24 hours or requires outpatient IV hydration | Emergency room visit or hospitalization |
| Vomiting | No interference with activity or 1-2 episodes/24 hours | Some interference with activity or > 2 episodes/24 hours | Prevents daily activity, requires outpatient IV hydration | Emergency room visit or hospitalization |
| Constipation | Change in stool softness or number of defecations | Decreased number of defecations with hard and dry stool, change in food intake, use of regular laxative or enema | Significant constipation in need of finger enema | - |
| Stomachache | No interference with activity | Some interference with activity not requiring medical intervention | Prevents daily activity and requires medical intervention | Emergency room visit or hospitalization |
| Rash | Erythema (itch) or local macule | Systemic macule, maculopapular rash, dry desquamation | Vesicular rash or moist desquamation or ulceration | - |
| Dizziness | No interference with activity | Some interference with activity | Prevents daily activity | - |
| Cough | Transient (not requiring medical intervention) | Continuous (requiring medical intervention) | Paroxysmal cough (uncontrollable with treatment) | - |
| Dyspnea | Dyspnea during exercise | Dyspnea during daily activity | Dyspnea during resting | - |
| Hypersensitivity | Transient erythema or rash | Rash, redness, urticaria, dyspnea | Symptomatic bronchospasm, use of non-oral treatment, allergic edema/angioedema, hypotension | - |
| Other | No interference with activity | Some interference with activity not requiring medical intervention | Prevents daily activity and requires medical intervention | Emergency room visit or hospitalization |

Grading criteria defined based on the Food and Drug Administration guidelines.^14^
