# Supplementary material for: Risk Factors for Grade 3 to Grade 4 Adverse Reactions to the ChAdOx1 nCoV-19 Vaccine (AZD1222) Against SARS-CoV-2
Source: Front Med (Lausanne). 2021 Sep 30;8:738049. doi: 10.3389/fmed.2021.738049 (PMC8514770; doi:10.3389/fmed.2021.738049)

Supplementary Figure 1. Solicited adverse reactions after the first dose of ChAdOx1 nCoV-19 by sex; AR, adverse reaction (F, female, n = 1,261; M, male, n = 342).

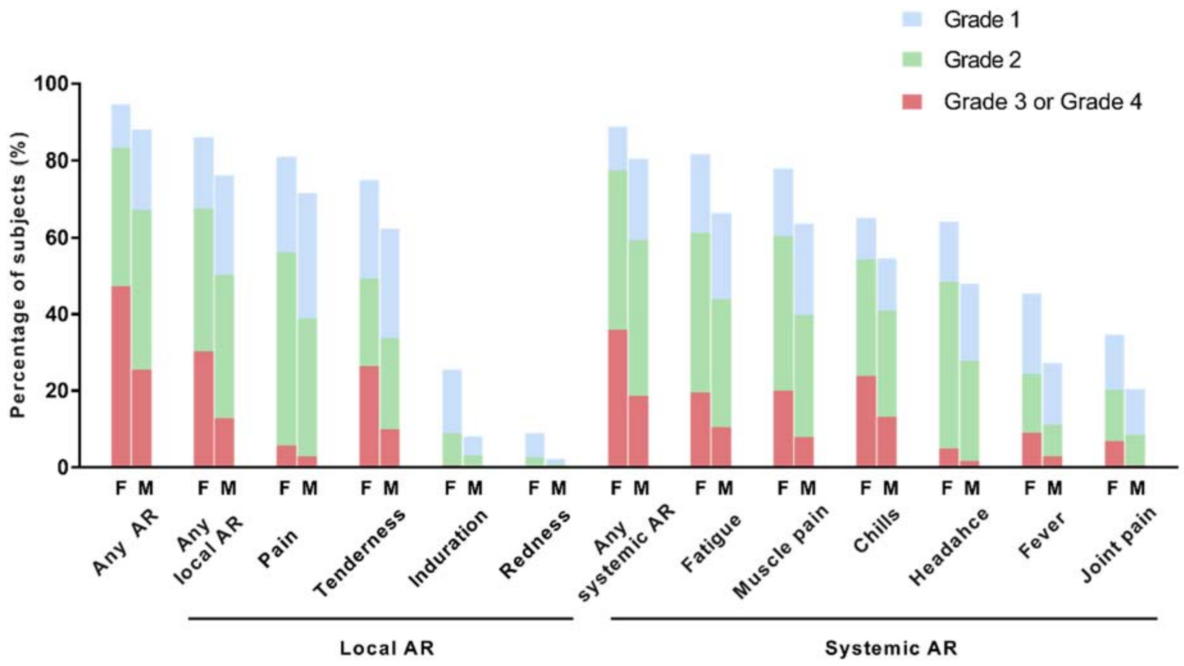

Supplement: Supplementary file 4 [file Data_Sheet_1.PDF]
