# Supplementary material for: Risk Factors for Grade 3 to Grade 4 Adverse Reactions to the ChAdOx1 nCoV-19 Vaccine (AZD1222) Against SARS-CoV-2
Source: Front Med (Lausanne). 2021 Sep 30;8:738049. doi: 10.3389/fmed.2021.738049 (PMC8514770; doi:10.3389/fmed.2021.738049)

Supplementary Figure 2. Solicited adverse reactions after the first dose of ChAdOx1 nCoV-19 by age; AR, adverse reaction (20–30 years, n = 588; 31–40 years, n = 319; 41–50 years, n = 468; >50 years, n = 228).

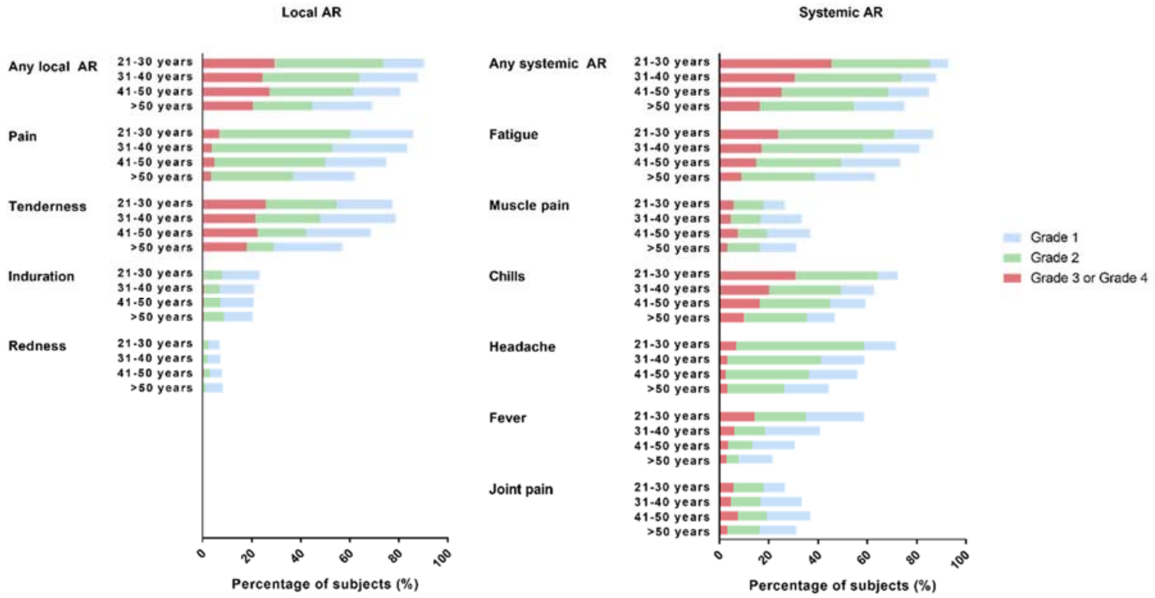

Supplement: Supplementary file 5 [file Data_Sheet_2.PDF]
