# Supplementary material for: Risk Factors for Grade 3 to Grade 4 Adverse Reactions to the ChAdOx1 nCoV-19 Vaccine (AZD1222) Against SARS-CoV-2
Source: Front Med (Lausanne). 2021 Sep 30;8:738049. doi: 10.3389/fmed.2021.738049 (PMC8514770; doi:10.3389/fmed.2021.738049)

Supplementary Figure 3. Solicited adverse reactions after first-dose of ChAdOx1 nCoV-19 by body mass index; AR, adverse reaction; BMI, body mass index (<18.5 kg/m^2, n = 121; 18.5-22.9 kg/m^2, n = 867; 23.0-24.9 kg/m^2, n = 292; ≥25 kg/m^2, n = 323).

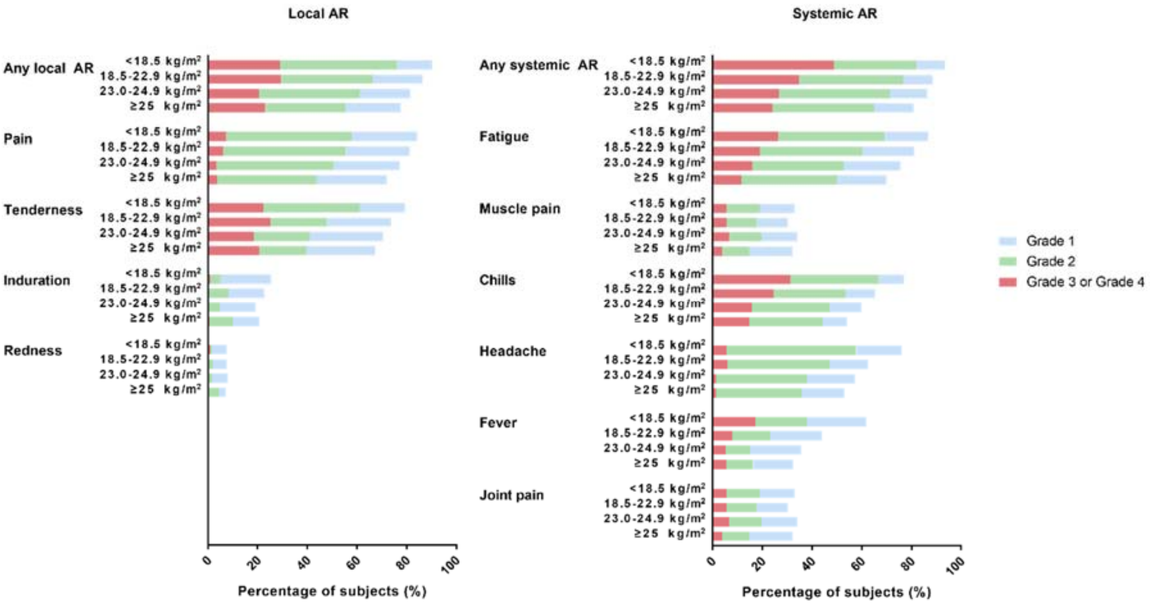

Supplement: Supplementary file 6 [file Data_Sheet_3.PDF]
